# EVALUATION OF ENEMAS CONTAINING SUCRALFATE IN TISSUE CONTENT OF MUC-2 PROTEIN IN EXPERIMENTAL MODEL OF DIVERSION COLITIS

**DOI:** 10.1590/0102-6720201700020012

**Published:** 2017

**Authors:** Oscar Orlando Araya FERNANDEZ, José Aires PEREIRA, Fábio Guilherme CAMPOS, Carolina Mardegan ARAYA, Gabriele Escocia MARINHO, Rafaela de Souza NOVO, Thais Silva de OLIVEIRA, Yara Tinoco FRANCESCHI, Carlos Augusto Real MARTINEZ

**Affiliations:** 1 Laboratory of Medical Research of the Post-Graduation Program in Health Sciences, São Francisco University, Bragança Paulista, SP, Brazil.

**Keywords:** Colon, Colitis, Sucralfate, Mucin-2, Fatty acids volatile

## Abstract

**Background:**

The effects of topical application of sucralfate (SCF) on the tissue content of MUC-2 protein have not yet been evaluated in experimental models of diversion colitis.

**Aim:**

To measure the tissue content of MUC-2 protein in the colonic mucosa diverted from fecal stream submitted to the SCF intervention.

**Methods:**

Thirty-six rats underwent derivation of intestinal transit through proximal colostomy and distal mucous fistula. The animals were divided into three groups which were submitted application of enemas with saline, SCF 1 g/kg/day and SCF 2 g/kg/day. Each group was divided into two subgroups, according to euthanasia was done after two or four weeks. The colitis diagnosis was established by histopathological study and the inflammatory intensity was evaluated by previously validated scale. The MUC-2 protein was identified by immunohistochemistry and the tissue content was measured computerized morphometry).

**Results:**

The application of enemas with SCF in the concentration of 2 g/kg/day reduced inflammatory score of the segments that were diverted from fecal stream. The content of MUC-2 in diverted colon of the animals submitted to the intervention with SCF, independently of intervention period and the used concentration, was significantly greater than animals submitted to the application of enemas containing saline (p< 0.01). The content of MUC-2 after the intervention with SCF in the concentration of 2 g/kg/day was significantly higher when compared to the animals submitted to the application containing SCF at concentration of 1.0 g/kg/day (p<0.01). The tissue content of MUC-2 reached the highest values after intervention with SCF in the concentration of 2 g/kg/day for four weeks (p<0.01). ***Conclusion:*** The preventive application of enemas containing SCF reduces the inflammatory score and avoids the reduction of tissue content of MUC-2, suggesting that the substance is a valid therapeutic strategy to preserve the mucus layer that covers the intestinal epithelium.

## RESUMO

**Racional:**

Os efeitos da aplicação tópica de sucralfato (SCF) no conteúdo tecidual da proteína mucina-2 (MUC-2) ainda não foram avaliados em modelos experimentais de colite de exclusão.

**Objetivo:**

Mensurar o conteúdo tecidual da proteína MUC-2 na mucosa cólica sem trânsito intestinal submetida à intervenção com SCF.

**Método:**

*:* Trinta e seis ratos foram submetidos à derivação intestinal por colostomia proximal terminal e fístula mucosa distal. Foram divididos em três grupos segundo recebessem clisteres contendo solução fisiológica (SF), SCF 1 g/kg/dia e SCF 2 g/kg/dia. Cada grupo foi dividido em dois subgrupos, segundo a eutanásia ser realizada após duas ou quatro semanas. O diagnóstico de colite foi estabelecido por estudo histopatológico e a intensidade inflamatória foi avaliada por escala validada. A expressão tecidual da MUC-2 foi identificada por imunoistoquímica e seu conteúdo mensurado por morfometria computadorizada.

**Resultados:**

A aplicação de clisteres com SCF na concentração de 2 g/kg/dia reduziu a intensidade inflamatória no cólon sem trânsito fecal. O conteúdo tecidual de MUC-2 no cólon sem trânsito dos animais submetidos à intervenção com SCF, independente do tempo de intervenção e da concentração utilizada, foi maior quando comparado aos animais tratados com SF (p<0,01). O conteúdo de MUC-2 após a intervenção com SCF na concentração de 2 g/kg/dia foi maior quando comparado aos animais submetidos à intervenção com concentração menor (p<0,01). O conteúdo de MUC-2 foi maior após intervenção com SCF na concentração de 2 g/kg/dia por quatro semanas (p<0,01).

**Conclusão:**

A aplicação preventiva de clisteres com SCF reduz o grau de inflamação e preserva o conteúdo tecidual de MUC-2, em segmentos desprovidos de trânsito intestinal, mostrando-se uma estratégia terapêutica válida para preservar a camada de muco que recobre o epitélio intestinal.

## INTRODUCTION

The large intestine epithelium constitutes the most perfect morphofunctional barrier of the human body, preventing the penetration of antigens, toxins and bacteria present in the lumen of the colon into the internal environment^3^ ^,^ ^5^ ^,^ ^27^. This barrier is composed of different lines of defense, mainly represented by the layer of mucus that covers the colonic mucosa; by a single layer of juxtaposed cells firmly adhered to each other that form the epithelial surface; by the efficient mechanisms of intercellular junctions that connect a cell to its neighbor, by the basement membrane; and by a complex immune defense system^9^ ^,^ ^28^. This immune system, considered the most efficient of the human organism, protects the host by hindering the translocation of pathogens and toxins from intestinal lumen. Therefore, the protection conferred by the different defense mechanisms of the colic epithelial barrier is essential for the preservation of human life.

The cells of the colonic epithelium are covered by a gelatinous layer of mucus that acts as the first line of defense of the epithelial barrier. Mucus, besides functioning as a lubricating agent facilitating the progression of the fecal content, has antibacterial properties and also selectively permeates the intestinal wall^9^ ^,^ ^11^ ^,^ ^33^. The mucus layer is predominantly composed of mucins, a class of glycoproteins that are constituents of its chemical composition and its mechanical barrier function^7^ ^,^ ^8^. Mucins are produced by goblet cells present in large quantities in the colic glands. Short chain fatty acids (SCFA) are the most important substrate for goblet cells to obtain sufficient energy to continuously produce mucins^1^ ^,^ ^10^ ^,^ ^11^ ^,^ ^36^.

The molecular structure of the mucin molecules is composed of a glycidic and a protein fraction. When considering its glucose fraction, it is subdivided into neutral mucins, more abundant in the upper digestive system, and the acidic ones, mainly found in the large intestine^26^. Acidic mucins, on the other hand, are subdivided into sulfomucines when there is predominance of sulphated radicals in their molecular structure or sialomucins when there is a higher content of sialic acid^7^ ^,^ ^21^ ^,^ ^26^. Regarding the protein fraction, there are more than 20 mucin subtypes described, but those of the MUC-2 subtype are the most abundant in the colonic epithelium. The MUC-1, MUC-3, MUC-4 and MUC-5 subtypes can also be found in the colonic mucosa, but in a smaller amount. In a similar way to the glucose fraction of the molecule, the adequate supply of GCFA plays an important role in the synthesis of glycoprotein protein fractions^2^ ^,^ ^4^ ^,^ ^8^. AGCCs have been shown to be capable of 20 fold increase in MUC-2 gene expression and, consequently, transcription of the mRNA responsible for the translation of the homologous protein^11^. These findings confirm the importance that the regular supply of GCFA has for the adequate synthesis of mucins by the goblet cells present in the intestinal epithelium^24^. Studies have shown that changes in the type and content of mucins that cover the colonic epithelium may occur in different inflammatory bowel diseases. Changes in the thickness and constitution of the mucus layer have been described in bacterial colitis, ulcerative colitis, exclusion colitis (EC), adenomatous polyps and colorectal cancer^3^ ^,^ ^5^ ^,^ ^7^ ^,^ ^8^ ^,^ ^21^ ^,^ ^26^. Thus, the study of the expression of the mucin and protein fractions is considered an important indicator of the functional integrity of the epithelial barrier of the colonic mucosa.

Experimental studies in EC models have shown that in colonic segments deprived of intestinal transit, where there is no adequate supply of GCFA, there is a reduction in the content and modifications in the expression pattern of the mucin glycemia fractions in inflamed colonic mucous glands^21^ ^,^ ^26^. These changes worsen with intestinal bypass time, reinforcing the importance of regular supply of GCFA for glycoprotein synthesis^17^ ^,^ ^21^ ^,^ ^26^. Differently, the application of solutions rich in SCFA or substances that protect the colonic mucosa from the action of antigens and bacteria present in the intestinal lumen and stimulate the production of mucus, are considered valid therapeutic strategies for the treatment of EC^3^ ^,^ ^5^ ^,^ ^19^.

Sucralfate (SCF) is a molecule composed of sucrose octassulfate and polyaluminium hydroxide. Due to its polyonic composition, it binds strongly with proteins of positive charges of the inflamed tissues forming an adherent complex that protects the mucosa from the harmful action of the digestive secretions and pathogenic bacteria present in the feces. This complex adhered to the inflamed epithelial surface hinders the penetration of external harmful agents into the internal environment^27^. SCF also presents other pharmacological properties, highlighting its antioxidant action and stimulating the production of epithelial growth factor, important in the cellular renewal processes^30^. Studies have shown that one of the main mechanisms of action of SCF is related to its capacity to increase the synthesis and release of prostaglandin E2 stimulating the production of mucus from the goblet cells^16^ ^,^ ^18^. The effects of application of enemas with SCF on the amount and pattern of expression of the mucin glycogenic portions in colonic mucous glands devoid of fecal transit, have already been studied in experimental models of EC. However, the tissue content of the mucin protein fraction has not yet been evaluated in experimental studies or in EC patients^3.5^.

Thus, the objective of the present study was to evaluate experimentally the effects of application of SCF-containing enemas on the tissue content of the MUC-2 protein in glands of colonic mucosa devoid of intestinal transit.

## METHOD

This research obeyed Federal Law 11.794 and the guidelines of the Brazilian College of Animal Experimentation. The research project was approved by the Ethics Committee on the Use of Animal in Research of the São Francisco University. The Project was approved with Nº. 002.04.10.

Thirty-six male Wistar rats from the São Francisco University Animal Hospital, Bragança Paulista, SP, Brazil, were used. The weight varied between 300-320 g and had an average of four months of age.

**FIGURE 1:**
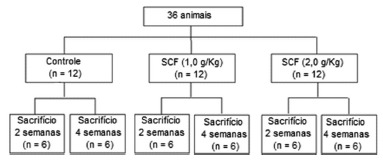
Algorithm of the division of experimental groups

Three study groups were randomly assigned to 12 animals each, divided according to the daily application of enemas containing a physiological solution (SF 0,9%), SCF solution at a concentration of 1 g/kg/day (SCF -1) and at the concentration of 2 g/kg/day (SCF-2), respectively. Six animals from each of these groups underwent euthanasia after two weeks of intervention, while the remaining 18, six from each experimental group, after four weeks (Figure 1).

During the preoperative period (seven days), they were isolated in individual cages, kept in air conditioned shelves, with temperature control, luminosity, humidity and noise. They fasted for 12 h, except for water, before surgery. Each cage was marked with the number of the animal, the group and the experimental subgroup to which it belonged. These same data were tattooed with ink on the tail of each animal.

On the day of the intervention, they were weighed to calculate the anesthetic dose to be used. Anesthesia was given using 2% xylazine hydrochloride and ketamine hydrochloride at a dose of 0.1 ml/100 g given intramuscularly to the left hind paw.

### Operative technique

After being anesthetized, they were placed on the surgical board, in a horizontal dorsal decubitus, and the entire abdominal region was trichotomized. Cutaneous antisepsis was performed with polyvinylpyrrolidone-iodine application on depilated area, later isolated by a sterile fenestrated surgical field. The laparotomy was performed by means of an infra-umbilical medial longitudinal incision with 3 cm extension. Once the abdominal cavity was opened, the cecum was identified and with the aid of a pachymeter the location chosen for the section of the right colon, located 4 cm after the ileocecal papilla, was measured in the large intestine. After ligation of the vessels of the marginal colonic arcade, the colon was sectioned at the predetermined point and the proximal large intestine was externalized as a terminal colostomy through a circular incision, 3 mm in diameter, located in the right iliac fossa. The colostomy was attached to the skin with separate stitches of absorbable 4-0 monofilament thread at the four cardinal points, and then between them, tying with three knots. After the preparation of the proximal stoma, the caudal segment of the sectioned large intestine was catheterized with a polyvinyl (12 F) probe. After, the catheterized colon was irrigated with 60 ml of physiological solution heated to 37° C, until the effluent drained by the anus did not present the output of any fecal content. After cleansing of the large intestine, the catheter was removed and the caudal segment of the colon externalized as a mucosal fistula located in the right hypochondrium. The mouth of the caudal stoma was fixed with the same technique used in the proximal colostomy. Abdominal wall synthesis was performed on two planes of suture: peritoneum and aponeurosis with continuous stitches of 4-0 polyglycolic acid thread and skin with 4-0 separate nylon stitches.

### Postoperative

After the operation, the animals were maintained for 10 min under heating by incandescent lamp. When they recovered the wake, they were housed in the individual cages previously identified, releasing the water intake and standard rodent ration (Nuvilab CR1®). They remained in individual cages until the date of euthanasia (two or four weeks), in the same environmental conditions of humidity, luminosity and temperature of the preoperative period. After the fecal flow bypass operation, no further care was taken with respect to operative wound or stomata. For the first three days after the surgical intervention for analgesia, paracetamol was used at a dosage of 200 mg/kg dissolved in the drinking water and given twice a day.

### Intervention with proposed solutions and material collection

The animals were submitted to the daily application of enemas with the standardized intervention solutions. The application was always performed using a polyethylene catheter graduated in centimeters with an internal diameter of 14 F. The catheter was carefully inserted through the anus at the standardized depth 3 cm from the anal border. Next, the enema containing the intervention solution proposed for each experimental group at room temperature was slowly applied until it drained through the distal mucous fistula (colostomy excluding fecal transit) located in the right hypochondrium.

On the eve of euthanasia (7 or 14 days), the animals were again weighed and fasted for 12 h, except for water. All were given the application of the enema with the intervention solution in the morning of the scheduled date for euthanasia always performed in the afternoon. For the removal of the segment of the large intestine to be studied, the animals were anesthetized with the same technique previously described, making a wider opening of the abdominal cavity. After release of the adhesions, if present, the entire colon devoid of fecal transit was removed. Still anesthetized the animals were sacrificed with lethal intracardiac dose of thiopental.

After removing the colic segments were carefully opened by the contramesenteric border. After being opened they were washed with 0.9% saline solution (SF) at 37° C for removal of remaining fecal residues. A longitudinal fragment measuring 30 mm length was removed, affecting the entire intestinal wall. This fragment was always removed from the derived colic segment, submitted to intervention solutions. A 10 mm segment of the colon was discarded from the stoma fixation to the skin, as well as another 2 cm above the anus, which included the anal canal. The collected fragment was submitted to histological study, using standard H&E staining and immunohistochemistry (primary antibody Anti-MUC2, Dako do Brasil, clone: ​​NCH-38) at 1:100 dilution and with positive control of MUC-2 made from the same technique described in tissue obtained from human colon, while the negative was performed on the same tissue, however without the addition of the primary antibody during the reaction.

### Histological evaluation of the presence of colitis

To confirm the histopathological diagnosis of colitis, the following parameters were considered: presence and number of ulcerations in the epithelium and intensity of inflammatory infiltrate, according to previously used and modified scale^3^ ^,^ ^5^. In this scale, the degree of tissue inflammation was evaluated according to the intensity of the neutrophilic infiltration in the mucosa and the degree of epithelial loss. The values ​​found were stratified into four grades: 0, when there was no neutrophil infiltration or epithelial loss; 1, when there was infiltration of neutrophils <50% of the colic glands without epithelial loss; 2, when there was neutrophil infiltration <50% of the colic glands and formation of up to two epithelial ulcers; 3, when there was infiltration of neutrophils ≥50% of the colic glands and formation of up to two epithelial ulcers; 4, when there was neutrophilic infiltration in ≥50% of the colic glands and formation of more than three epithelial ulcers.

### Measurement of tissue content of MUC-2

Two slides were analyzed for each animal. Expression of the MUC-2 protein was studied according to the site and content of immunostaining in the colonic glands. The selected image, after properly focused, was captured by a camcorder coupled to the optical microscope. The tissue content of the protein was measured by computer-aided image analysis (computerized morphometry). The captured image was processed and analyzed by the NIS-Elements® program (Nikon do Brasil Ltda., São Paulo) installed in a microcomputer with good image processing capacity. The content of MUC-2 was quantified in each of the two prepared slides in three adjacent and complete colonic crypts present in three distinct histological fields. Thus, for each animal the tissue content of MUC-2 was quantified in 18 colic glands (nine on each prepared slide).

Protein quantification was always performed after calibration of the program for the magnification selected in the microscope, always redone after reading each slide. For the quantification of the color density found in each selected field, we used a RGB filter, adopting any wavelength that contained the whole spectrum of brown color (color that identified the tissue immunoexpression of the MUC-2 protein). With the program, the staining was transformed with white immunoexpression and the rest of the field of view captured, without immunostaining, in black composing a binary image. The values ​​found for the tissue content of MUC-2 were always expressed as percentage by field analyzed (%/field). The final value for the control and experimental animals (SF, SCF-1 and SCF-2) was always represented by the mean reading of the two slides (18 colic glands), with the respective standard error. All selected images have been archived for further scientific documentation.

### Statistical analysis

The results obtained after reading were always described by the mean with respective standard error. A significance level of 5% (p<0.05) was adopted for all tests. The Mann-Whitney test was used to analyze the degree of inflammation and tissue content of the MUC-2 protein, comparing the animals from the control and experiment groups. The ANOVA test was used to analyze the variance of the tissue content of the MUC-2 protein in relation to the intervention time. For the statistical study the BioStat program (version 5.1) was used. Significant values ​​when the irrigated segments were compared with SF and SCF (1 g/kg/day or 2 g/kg/day) were marked with an asterisk (*) when the level of significance was lower than 5%, and two asterisks (**) when this level was less than 1%. In the same way, the significant values ​​found when the animals submitted to the intervention with SCF at the lowest concentration (1 g/kg/day) and those submitted to irrigation with a higher concentration (2 g/kg/day) were marked with a ticket (•) when the level of significance was less than 5% or two (••) when less than 1%.

## RESULTS

**FIGURE 2:**
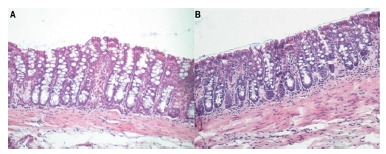
A) Histological section of the colonic mucosa derived after irrigation with SF for four weeks (H&E-100x); B) histological section of the colonic mucosa derived after irrigation with SCF at the concentration of 2 g/kg/day for four weeks. A thin film of SCF covering the surface of the mucosa (H&E-200 x) is observed.

Figure 2A shows the segment obtained from the SF-irrigated colon for four weeks, while Figure 2B shows the colon irrigated with SCF at 2.0 g/kg/day for the same period of time. It is verified that in animals of the control group there is a clear epithelial loss, disarrangement in the architecture and in the alignment of the colic glands, whereas in the SCF 2 g/kg/day the epithelial surface is preserved, intestinal crypts present aligned with normal distribution pattern and preservation of the integrity of goblet cells. It is possible to observe a thin eosinophilic SCF film covering the epithelial surface of the colonic mucosa.

Table 1 shows the degree of inflammation found in the segments devoid of fecal transit after SF, SCF 1 g/kg/day and 2 g/kg/day for two and four weeks. The results show that the SCF intervention at 2 g/kg/day in the derived colic segment reduced the degree of inflammation after four weeks of irrigation when compared to the control group (p=0.03).

**TABLE 1:** Inflammatory score in the colic segment derived from the animals submitted to intervention with SF, SCF-1 (SCF 1 g/kg/day) and SCF-2 (2 g/kg/day) submitted to intervention for two and four weeks.

Mean±SD				
	S.F. 0,9%	SCF 1 g/kg/day	SCF 2 g/kg/day	p
2 weeks	3.5±0.25	3.3±0.32	3.1±0.29	NS
4 weeks	3.1±0.20	2.8±0.25	2.4±0.31*	0.03

*=p<0,05 (SCF 2,0 g/kg/day × S.F.0,9%); Mann-Whitney test

**FIGURE 3:**
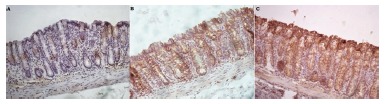
A) Histological section of a colonic segment where there is a reduction of MUC-2 expression after irrigation with SF for four weeks (100x); B) colonic segment, where higher MUC-2 expression was observed after irrigation with SCF at the concentration of 1 g/kg/day for four weeks when compared to irrigation with SF (100x); C) derived colic segment where MUC-2 expression was increased on the epithelial surface after irrigation with SCF at a concentration of 2 g/kg/day for four weeks when compared to animals irrigated with SF and SCF 1 g/kg/day (100x).

Figure 3A shows the tissue expression of MUC-2 in the colon irrigated with SF for four weeks and to 3B the SCF-irrigated colon at the concentration of 1 g/kg/day for the same period of time. Figure 3C shows colon treated with SCF at a concentration of 2 g/kg/day for four weeks. It is verified that in the animals submitted to SF intervention there is a reduction in MUC-2 content in goblet cells, architecture derangement and alignment of the colic glands. Undergoing SCF intervention at 1 and 2 g/kg/day the MUC-2 content is significantly more evident in goblet cells. In animals treated with SCF concentration of 2 g/kg/day, a greater increase in MUC-2 content was observed in goblet cells mainly located on the luminal surface of the colic glands

Figure 5 compares the MUC-2 content found in segments devoid of fecal transit after SF, SCF 1 g/kg/day and 2 g/kg/day, for two and four weeks. The results showed that SCF intervention, at a concentration of 1 g/kg/day and 2 g/kg/day in the derived colic segment, allowed significantly higher content of MUC-2 after two and four weeks of irrigation when compared to the animals of the group control. The highest content of MUC-2 found is related to the dose of SCF administered (p<0.01).

**FIGURE 4:**
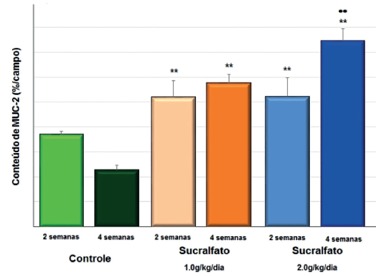
Tissue content of MUC-2 in control, SCF-1 and SCF-2 animals submitted to daily intervention with SCF for two and four weeks

**=p<0,01 (SCF-1 × control; SCF-2 × control); ••=p<0,01 (SCF-1 × SCF-2); Mann- Whitney test

Table 2 shows the variation in MUC-2 content according to the SCF concentration used and the time of use of the SCF, relative to the control group. The content of the neutral and acidic mucins in the experimental groups was higher than the control group, at concentrations of 1 g/kg/day and 2 g/kg/day SCF, and in both intervention periods evaluated (p<0.01) .

**TABLE 2:** Variation in the mean tissue content of MUC-2 according to the intervention time in the animals submitted to intervention with SF, SCF 1 g/kg/day and 2 g/kg/day for two and four weeks of intervention

Mean±SD					
SF		SCF 1 g/kg/day		SCF 2 g/kg/day	
2 weeks	4 weeks	2 weeks	4 weeks	2 weeks	4 weeks
3.68±0.14	2.27±0.20	5.20±0.65	5.76±0.35	5.22±0.75	7.45±0.49**

SF=physiological solution 0.9%; SCF=sucralfate; **=significant p<0.01; ANOVA test

## DISCUSSION

AGCCs are formed in the colon from the bacterial fermentation of carbohydrates ingested in the diet that escaped the hydrolysis in the small intestine. Acetate, propionate and butyrate are the most commonly produced subtypes within the large intestine. SCFAs have several functions in the large intestine, since they reduce intestinal pH, stimulate cell proliferation, and increase blood flow to the colonic mucosa. ACGG also represent the main energy substrate necessary for the different stages of cellular metabolism^4^. The proportion of different types of SCFA in the large intestine is determined by the type of feed, as well as by the composition of the intestinal microbiota which, in turn, is also influenced by the diet ingested. Prebiotics stimulate the proliferation and activity of bifidobacteria within the colon that greatly interfere with the composition and luminal content of AGCC^12^. Butyrate represents the main source of energy for the cells of the colic epithelium. The substance has a prominent antitumorigenic effect in many tumor cell lines, plays a significant role in the maintenance of colonic mucosal homeostasis, and regulates the expression of genes responsible for the processes of proliferation, differentiation and apoptosis^6^. In this way, butyrate presents fundamental importance for the maintenance of health in the colonic mucosa. Any changes in its intraluminal production or in its supply are associated with the development of a series of diseases of the large intestine^35^.

EC is characterized by the development of an inflammatory process in the mucosa of segments of the large intestine, excluded from fecal transit^13^. Most of the authors attribute its occurrence to the deficiency in the production of CCFA in the intestinal lumen, caused by the absence of substrate supply and reduction of the microbiota responsible for its synthesis^37^. This possibility is reinforced when it is observed that the reconstitution of the transit, or irrigation of the excluded segments with nutrient solutions rich in CCFA, restoring the supply of the main energy substrate, are able to revert to the clinical and histopathological changes found in patients with EC^25^ ^,^ ^37^.

The molecular mechanisms by which the deficiency in the normal supply of GCFA leads to the appearance of EC appear to be related to the increase in the production of oxygen free radicals (RLO), by the epithelial cells themselves deprived of their main energy source^22^. Without the main energy fuel epithelial cells undergo changes in their cellular energy metabolism causing increased production of RLO. The resulting oxidative stress causes a breakdown of the defense mechanisms that form the colonic mucosa, allowing the infiltration of bacteria and antigens in the sterile layers of the colonic wall, allowing the development of EC. Studies have shown that RLOs damage the different defense systems that form the colonic mucosal barrier^19^ ^,^ ^21^ ^,^ ^26^ ^,^ ^28^.

The mucus layer that covers the colic epithelium is a critical component of these defense mechanisms, as it provides mechanical protection, creating an interface between the intestinal bacteria and the colic epithelium. It has an antimicrobial effect conferred by the peptides present in the mucin molecules. Experimental studies have shown that the lack of supply of CCFA and the consequent tissue oxidative stress causes significant changes in the content and pattern of mucin distribution in the intestinal mucosa^17^ ^,^ ^21^ ^,^ ^26^. These studies, when evaluating the tissue content of the glucose fractions of the mucin molecule, found a marked reduction in the tissue content of the neutral and acidic mucins in the glands of the excluded colon when compared to the colon with the preserved fecal transit^17^ ^,^ ^26^. They also showed that the reduction in the tissue content of acid mucins was mainly due to the lower production of sialomucins^21^. It is probable that the decrease in the tissue content of mucins in the colonic mucosa without fecal transit may be related to two different reasons. Firstly, by the decrease in the transcription of genes related to the production of the protein fraction of the glycoprotein molecule, as a consequence of the deficiency in the delivery of AGCC^4^ ^,^ ^10^ ^,^ ^11^. Second, by the destruction of the mucus layer that forms the first defense system of the epithelial barrier due to the increase in the production of RLO by the epithelial cells with alterations of its habitual energetic metabolism^19^ ^,^ ^22^. These possibilities are reinforced by showing that the addition of AGCC, in particular the butyrate, in cell culture or experimental models of EC induces the expression of the MUC-2 gene, increasing the production of the glycoprotein, besides reducing the tissue levels of RLO and, consequently, the damage to the colonic mucous epithelium^4^ ^,^ ^10^ ^,^ ^11^ ^,^ ^15^ ^,^ ^19^ ^,^ ^29^. AGCCs also have additional benefits in preserving epithelial integrity, inhibiting the formation of proinflammatory cytokines inducing κB nuclear factor activation. They also act as antioxidants by increasing the production of catalase an important enzyme in neutralizing overproduced RLO. The CCFA also present an anti-inflammatory action by modulating the production of cicooxygenase-229. These effects make the administration of CLCC-containing enemas an effective complementary strategy for the treatment of different forms of colitis^14^ ^,^ ^34^.

When considering the different mechanisms involved in the etiopathogenesis of EC, the ideal substance for the treatment of the disease should have as properties the ability to adhere vigorously to the inflamed epithelium making bacterial infiltration difficult; stimulate mucin production by goblet cells; induce the expression of genes related to the transcription of the protein fraction of the molecule and to have antioxidant and anti-inflammatory action^27^. When these properties are considered, the SCF shows an interesting molecule to be tested in the treatment of EC. Recently, experimental studies have shown that the application of enemas with SCF in addition to forming a protective film, firmly adhered to the colonic mucosa, reduced the intensity of the inflammatory process in the mucosa devoid of fecal transit^27^. The improvement of the inflammatory process was shown to be related to the anti-inflammatory, antioxidant and stimulant action of the mucus production that SCF has^3^ ^,^ ^5^ ^,^ ^23^. The SCF application also reduced tissue RLO levels by increasing mucosal layer thickness and tissue content of the mucin molecule’s glucose fractions in the colonic glands. However, in spite of the fact that the use of SCF has been shown to increase the constituents of the glucose fraction of the mucin molecule in the colon devoid of intestinal transit, no study has evaluated the effect of the substance on the tissue content of the protein fraction of the molecule, particularly the mucin found in the colonic mucosa, MUC-2.

The results found in the present study seem to confirm the benefits of administration of SCF-containing enemas for EC treatment. It has been shown that the daily administration of SCF enemas reduced the degree of tissue inflammation, especially when used in higher concentrations and for a longer period of intervention. The animals submitted to the SCF intervention showed less neutrophilic infiltration, as well as better alignment of the cells of the mucosal surface and less formation of epithelial ulcers. In some animals submitted to SCF intervention, regardless of the concentration used or the time of application, it was possible to identify the formation of an eosinophilic film on the epithelial surface facing the organ lumen confirming the substance’s ability to adhere firmly to the injured epithelium. All these findings are in agreement with those found in previously published studies^3^ ^,^ ^5^.

With respect to the MUC-2 content in the mucosa excluded from fecal transit, the present study showed that the daily application of clones containing SCF, regardless of the dose used, significantly increased the tissue quantity of MUC-2 when compared to the animals submitted to the intervention with SF. The increase in the tissue content of MUC-2 was more evident when using enemas with higher concentration of SCF for a longer period of time. The animals submitted to the SCF intervention presented greater expression of the MUC-2 protein in the goblet cells present in all extension of the colic glands, and especially in those located on the epithelial surface when compared to the animals of the control group. These results are in agreement with those of previous studies showing that the SCF intervention in the mucosa excluded from the submitted fecal transit increased the content of the glucose fractions of the molecule^3^ ^,^ ^5^. Studies have shown that SCF administration increased the thickness of the mucus layer that recovers the gastrointestinal tract by 8%, increasing the tissue content of sulfomucines by 63%, and by 81% that of sialomucines, precisely the subtype of reduced mucin in the epithelium without fecal transit^31^. Therefore, with the findings found in the present study it is reasonable to assume that the SCF intervention is able to increase both fractions of the mucin molecule.

The reasons why the application of SCF increases the tissue content of mucins are still poorly understood. It is possible that the effects of SCF on the production of MUC-2 are related to the various pharmacological properties of the drug. Study has shown that SCF stimulates mucus production in goblet cells by increasing the production of prostaglandin E2^16^ ^,^ ^30^. It has also been demonstrated that the antioxidant activity of SCF, by removing RLO produced in excess by intestinal mucosa cells devoid of intestinal transit, preserves the integrity of the different defense systems of the colonic mucosa, particularly the mucus layer^20^ ^,^ ^23^. An experimental study reported that the antioxidant activity of SCF reduces the levels of phospholipid oxidation present in the membranes of cells of the colonic mucosa, decreasing apoptosis and, consequently, preserving the integrity of the epithelial barrier^23^. It is possible that the SCF also increases the transcription of the MUC-2 gene related to the production of the homonymous protein in the colon epithelium without fecal transit. The results of the present study, showing an increase in the tissue content of the MUC-2 protein, suggest that this phenomenon may be occurring. However, this possibility can only be confirmed after performing studies that evaluate the expression of the MUC-2 gene in the transit-excluded epithelium submitted to SCF intervention.

The results found in the present study showed that the application of enemas with SCF was able to maintain the tissue content of MUC-2 in the goblet cells present in the colic glands. We also confirmed the results of previous studies demonstrating that the application of enemas with SCF preserves integrity and reduces the degree of inflammation in the epithelium excluded from traffic. These properties, coupled with the bioavailability of the substance, its low cost and small incidence of side effects, may make the drug a new drug option for EC treatment, as well as other inflammatory bowel diseases^32^.

## CONCLUSION

The preventive application of SCFs reduces the degree of inflammation and preserves the tissue content of MUC-2 in segments devoid of fecal transit.

## References

[1] Augenlicht L, Shi L, Mariadason J, Laboisse C, Velcich A (2003). Repression of MUC-2 gene expression by butyrate, a physiological regulator of intestinal cell maturation. Oncogene.

[2] Awad AB, Kamei A, Horvath PJ, Fink CS (1995). Prostaglandin synthesis in human cancer cells influence of fatty acids and butyrate. Prostaglandins Leukot Essent Fatty Acids.

[3] Bonassa CEG, Pereira JA, Campos FGCM, Rodrigues MR, Sato DT, Chaim FDM, Martinez CAR (2015). Tissue content of sulfomucins and sialomucins in the colonic mucosa, without fecal stream, undergoing daily intervention with sucralfate. Acta Cir Bras.

[4] Burger-van Paassen N, Vincent A, Puiman PJ, van der Sluis M, Bouma J, Boehm G, van Goudoever JB, van Seuningen I, Renes IB (2009). The regulation of intestinal mucin MUC2 expression by short-chain fatty acids: implications for epithelial protection. Biochem J.

[5] Chaim FDM, Sato DT, Rodrigues MR, Dias AM, Silveira PP, Pereira JA, Martinez CAR (2014). Evaluation of the application of enemas containing sucralfate in tissue content of neutral and acid mucins in experimental model of diversion colitis. Acta Cir Bras.

[6] Daly K, Shirazi-Beechey SP (2006). Microarray analysis of butyrate regulated gene in colonic epithelial cells. DNA Cell Biol.

[7] Deplancke B, Gaskins HR (2001). Microbial modulation of innate defense goblet-cells and the intestinal mucus layer. Am J Clin Nutr.

[8] Finnie IA, Dwarakanath AS, Taylor BA, Rhodes JM (1995). Colonic mucins synthesis is increased by sodium butyrate. Gut.

[9] Gaudier E, Hoebler C (2006). Physiological role of mucins in the colonic barrier integrity. Gastroenterol Clin Biol.

[10] Gaudier E, Jarry A, Blottière HM, De Coppet P, Buisine MP, Aubert JP, Laboisse C, Cherbut C, Hoebler C (2004). Butyrate specifically modulates MUC gene expression in intestinal epithelial goblet cells deprived of glucose Am. J. Physiol. Gastrointest Liver Physiol.

[11] Gaudier E, Rival M, Buisine MP, Robineau I, Hoebler C (2009). Butyrate enemas upregulate MUC genes expression but decrease adherent mucus thickness in mice colon. Physiol Res.

[12] Gibson GR, Roberfroid MB (1995). Dietary modulation of the human colonic microbiota introducing the concept of prebiotics. J. Nutr.

[13] Glotzer DJ, Glick ME, Goldman H (1981). Proctitis and colitis following diversion of fecal stream. Gastroenterology.

[14] Hamer HM, Jonkers DM, Vanhoutvin SA, Troost FJ, Rijkers G, de Bruïne A, Bast A, Venema K, Brummer RJ (2010). Effect of butyrate enemas on inflammation and antioxidant status in the colonic mucosa of patients with ulcerative colitis in remission. Clin Nutr.

[15] Hatayama H, Iwashita J, Kuwajima A, Abe T (2007). The short chain fatty acid, butyrate, stimulates MUC2 mucin production in the human colon cancer cell line, LS174T. Biochem Biophys Res Commun.

[16] Hollander D, Tarnawski A, Gergely H, Zipser RD (1984). Sucralfate protection of the gastric mucosa against ethanol-induced injury a prostaglandin-mediated process?. Scand J Gastroenterol Suppl.

[17] Keli E, Bouchoucha M, Devroede G, Carnot F, Ohrant T, Cugnenc PH (1997). Diversion related experimental colitis in rats. Dis Colon Rectum.

[18] Konturek SJ, Kwiecien N, Obtulowicz W, Kopp B, Oleksy J (1986). Double blind controlled study on the effect of sucralfate on gastric prostaglandin formation and microbleeding in normal and aspirin treated man. Gut.

[19] Lameiro TMM, da Silva CMG, Marques LHS, Cunha FL, Almeida MG, Pereira JA, Martinez CAR (2011). Effects of butyrate on levels of lipid peroxidation in cells of the colonic mucosa without fecal stream: experimental study in rats. Rev bras. colo-proctol.

[20] Laudanno OM, Bedini OA, Cesolari JA, San Miguel P (1990). Evidence of anti-oxidant role of sucralfate in gastric mucosal protection. Ital J Gastroenterol.

[21] Martinez CAR, Nonose R, Spadari APP, Máximo FR, Priolli DG, Pereira JA, Margarido NF (2010). Quantification by computerized morphometry of tissue levels of sulfomucins and sialomucins in diversion colitis in rats. Acta Cir Bras.

[22] Martinez CAR, Ribeiro ML, Gambero A, Miranda DDC, Pereira JA, Nadal SR (2010). The importance of oxygen free radicals in the etiopathogenesis of diversion colitis in rats. Acta Cir Bras.

[23] Martinez CAR, Rodrigues MR, Sato DT, da Silva CMG, Kanno DT, Mendonça RLS, Pereira JA (2015). Evaluation of the anti-inflammatory and antioxidant effects of the sucralfate in diversion colitis. J. Coloproctol. (Rio J.).

[24] Mello RO, Silva CM, Fonte FP, Silva DL, Pereira JA, Margarido NF, Martinez CA (2012). Evaluation of the number of goblet cells in crypts of the colonic mucosa with and without fecal transit. Rev Col Bras Cir.

[25] Nassri CGG, Nassri AB, Favero E, Rotta CM, Martinez CAR, Margarido NF (2008). Influence of irrigation of nutritional solutions in the colon excluded of fecal stream. Experimental study in rats. Rev bras colo-proctol.

[26] Nonose R, Spadari APP, Priolli DG, Máximo FR, Pereira JA, Martinez CAR (2009). Tissue quantification of neutral and acid mucins in the mucosa of the colon with and without fecal stream in rats. Acta Cir Bras.

[27] Pereira JA, Rodrigues MR, Sato DT, Silveira PP, Dias AM, Silva CG, Martinez CAR (2013). Evaluation of sucralfate enema in experimental diversion colitis. J. Coloproctol (Rio J.).

[28] Pravda J (2005). Radical induction theory of ulcerative colitis World J. Gastroenterology.

[29] Sauer J, Richter KK, Pool-Zobel BL (2007). Physiological concentrations of butyrate favorably modulate genes of oxidative and metabolic stress in primary human colon cells. J Nutr Biochem.

[30] Scheiman JM, Kraus ER, Yoshimura K, Boland CR (1992). Effect of sucralfate on components of mucosal barrier produced by cultured canine epithelial cells in vitro. Dig Dis Sci.

[31] Slomiany BL, Piotrowski J, Tamura S, Slomiany A (1991). Enhancement of the protective qualities of gastric mucus by sucralfate role of phosphoinositides. Am J Med.

[32] Sobrado CW, Sobrado LF (2016). Management of acute severe ulcerative colitis: a clinical update. Arq Bras Cir Dig.

[33] Swidsinski A, Loening-Baucke V, Theissig F, Engelhardt H, Bengmark S, Koch S, Lochs H, Dörfel Y (2007). Comparative study of the intestinal mucus barrier in normal and inflamed colon. Gut.

[34] Tong LC, Wang Y, Wang ZB, Liu WY, Sun S, Li L, Su DF, Zhang LC (2016). Propionate ameliorates dextran sodium sulfate-induced colitis by improving intestinal barrier function and reducing inflammation and oxidative stress. Front Pharmacol.

[35] Wachtershauser A, Stein J (2000). Rationale for the luminal provision of butyrate in intestinal diseases. Eur J Nutr.

[36] Willemsen LE, Koetsier MA, Van Deventer SJ, Van Tol EA (2003). Short chain fatty acids stimulate epithelial mucin 2 expression through differential effects on prostaglandin E1 and E2 production by intestinal myofibroblasts. Gut.

[37] Wong JM, de Souza R, Kendall CW, Emam A, Jenkins DJ (2006). Colonic health fermentation and short chain fatty acids. J Clin Gastroenterol.

